# Agarose gel electrophoresis determination of bovine lipoproteins compared with a wet chemistry method

**DOI:** 10.3168/jdsc.2022-0223

**Published:** 2022-07-14

**Authors:** E. Behling-Kelly, C. Wong

**Affiliations:** 1 College of Veterinary Medicine, Cornell University, Ithaca, NY 14850; 2 Department of Biomedical Informatics, Harvard University, Cambridge, MA 02138

## Abstract

Quantification of serum lipoproteins provides information relative to the overall metabolic health, degree of lipid mobilization, and hepatic function of dairy cattle. Automated assays performed on benchtop chemistry analyzers and commercially available kits use reagents developed for human lipoproteins. The substantial physical and chemical differences between bovine and human lipoproteins potentially confounds the use of these assays in evaluating bovine lipoproteins. In this study, we prospectively analyzed serum lipoproteins from 56 Holstein cows using horizontal slab agarose gel electrophoresis to semi-quantify the high-density lipoprotein (HDL) and low-density lipoprotein (LDL) fractions by optical densitometry. Ultracentrifugation was used to confirm the electrophoretic separation pattern of the lipoproteins. The values obtained using the electrophoretic method were compared with values obtained by direct measure of HDL cholesterol, total cholesterol, and triglyceride (TG) concentrations on a Roche chemistry analyzer, and calculated LDL cholesterol. Correlation between these methods was poor for HDL (Passing-Bablok regression line: y = 30.31 + 0.853x) and could not be calculated for LDL. Automated HDL values were equal to, or higher than, the total cholesterol concentration in 25 of the 56 samples. The TG concentrations were above the reference interval in 18 samples, and these samples had an average of 96% of the cholesterol measured as HDL by the automated method, and 78% HDL by electrophoresis. Given that it is physiologically impossible to have more cholesterol within the HDL fraction than in the total serum fraction, and the increased proportion of TG found in LDL and very-low-density lipoprotein, our results draw into question the accuracy of the Roche automated assay in quantifying bovine lipoprotein fractions.

## Highlights

• Bovine lipoproteins are chemically divergent from human lipoproteins.
• Enzymatic assays for measurement of human HDL do not transfer well to bovine HDL.
• Electrophoresis identifies low-density lipoprotein in samples, whereas automated assays are inaccurate.

## Graphical Abstract

**Figure:**
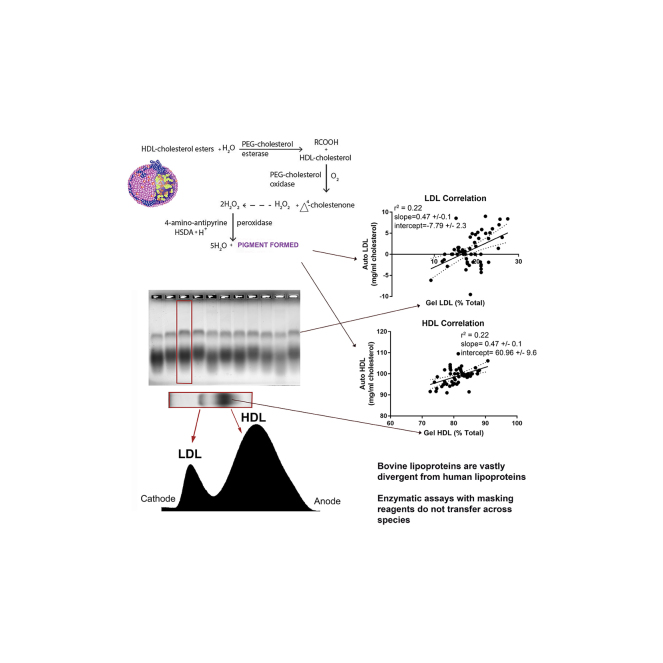
**Summary:** Automated assays for quantification of high-density lipoproteins (HDL) contain reagents that enzymatically react with intact HDL to generate a colored product that is proportional to the amount of cholesterol in the lipoprotein. The cholesterol esterase and oxidase in the assay reagent are polyethylene glycol modified (PEG), and thus have an increased affinity for HDL. The reagents mix also contains magnesium ions and dextransulfate, which form water-soluble complexes with lower density lipoproteins. The specificity of the reagents for HDL depends on the chemical composition of the lipoproteins, which is highly variable across species. In this study, we demonstrated that one of the commercially available reagents performed poorly in the quantification of bovine HDL, compared with an electrophoretic method.

Lipoproteins are macromolecular complexes composed of a triglyceride (**TG**) and cholesterol ester core surrounded and stabilized by phospholipids, unesterified cholesterol, and proteins. The major classes of lipoproteins in the cow are separated by density and include chylomicrons, very-low-density lipoprotein (**VLDL**), intermediate density lipoprotein, low-density lipoprotein (**LDL**), and high-density lipoprotein (**HDL**). The gold standard method of quantification of lipoproteins is ultracentrifugation, which is time consuming, laborious, and impractical for routine diagnostic testing (Chapman, 1980, 1986). Automated assays performed on benchtop chemistry analyzers and commercially available kits use reagents developed for the quantification of human lipoproteins. These assays typically rely on 1 of 2 basic methodologies. The first method uses repeated enzymatic measure of cholesterol before and after chemical precipitation of the LDL lipoprotein particles. In the second method, the reagents include polyanions or detergents (or both) to selectively solubilize LDL, VLDL, and chylomicrons. Thus, only HDL are left structurally able to react with the enzymatic detection steps for cholesterol and cholesterol esters that occur in the second step of the assay.

There are marked differences in the hydrated density and chemical composition of the individual lipoprotein classes across cattle and people. The metabolism of the bovine, presumptively ruminal hydrogenation, results in lipids that are significantly more saturated than in people (Nichols, 1967). Bovine HDL has a lighter hydrated density, more surface-exposed protein content, a higher proportion of cholesteryl ester and phospholipids, and an overall unique lipid content compared with human HDL (Evans et al., 1961; Jonas, 1972, 1973; Stead and Welch, 1976). Despite the documented differences in the physical properties and composition of bovine HDL compared with human HDL, a large number of studies investigating the effect of diet, reproductive stage, and lactational status on HDL concentration in cattle provide no primary nor cited method validation data (Gleockler et al., 1980; Turk et al., 2005, 2013; Mohebbi-Fani et al., 2006; Kessler et al., 2014; Gross et al., 2015; Saleem et al., 2016; Wu et al., 2020).

In this study, we hypothesized that wet chemistry methods developed for use on human samples would not be accurate in cattle. To test this hypothesis, we compared one wet chemistry method developed to measure human HDL (Roche Hitachi Modular P) to an electrophoretic method. Electrophoresis was selected as the comparative method because it physically separates bovine LDL and HDL in their native state and can be easily applied to many samples. Density gradient ultracentrifugation was used to confirm the electrophoretic separation pattern of the bovine lipoproteins.

Serum samples submitted to the Animal Health Diagnostic Center (Ithaca, NY) for diagnostic purposes were selected for inclusion based on total cholesterol concentrations measured using a cholesterol esterase/oxidase colorimetric assay (Roche). A total of 56 samples were identified, all from adult Holstein cattle. Residual samples were used within 48 h for lipoprotein electrophoresis and biochemical analysis including triglyceride concentration, a re-run of total cholesterol concentration, and measurement of HDL concentration using Roche's third generation HDL reagent on a Hitachi ModP analyzer. The TG concentrations were measured using a reagent that includes a lipase enzyme to hydrolyze the bonds between the glycerol and fatty acid chains, coupled to enzymatic and colorimetric detection of the freed glycerol backbone (Roche). Both total cholesterol and triglyceride assays were performed in accordance with the standard operating procedures of the Cornell Veterinary Clinical Pathology laboratory after daily quality control confirmation of assay performance. The LDL cholesterol concentration was calculated. Separate blood samples were collected for the isolation of lipoproteins by ultracentrifugation into EDTA tubes from cows at the Cornell University Teaching Dairy under Animal Care and Use Protocol 2007–0146.

Each serum sample (15 µL) was prepared by addition of 5 µL of loading buffer (60% sucrose, 0.1% bromophenol blue in double-distilled H_2_0) and loaded by volume (15 µL) into a 1% agarose gel (Agarose Unlimited) prepared in 60 m*M* sodium-barbital buffer (Sigma-Aldrich). Lipoproteins were separated by horizontal electrophoresis at 80 V for 55 min (BioRad) in 60 m*M* sodium-barbital buffer running buffer (Sigma-Aldrich). Gels were stained overnight at room temperature (20–22**°**C) in 0.18% Sudan black B (Sigma-Aldrich) in 70% ethanol. Gels were destained in a 15% acetic acid/20% acetone solution in double-distilled H_2_O for 2 to 3 h, until the background was light gray to clear and the bands were clearly visible. Gels were scanned (Epson Perfection V500) as negative images, and lipoprotein bands were quantified by converting the pixel intensity of the scanned lane to a linear peak plot followed by calculation of the area under the curve for each defined peak using National Institutes of Health Image J software (Figure 1).

**Figure 1:**
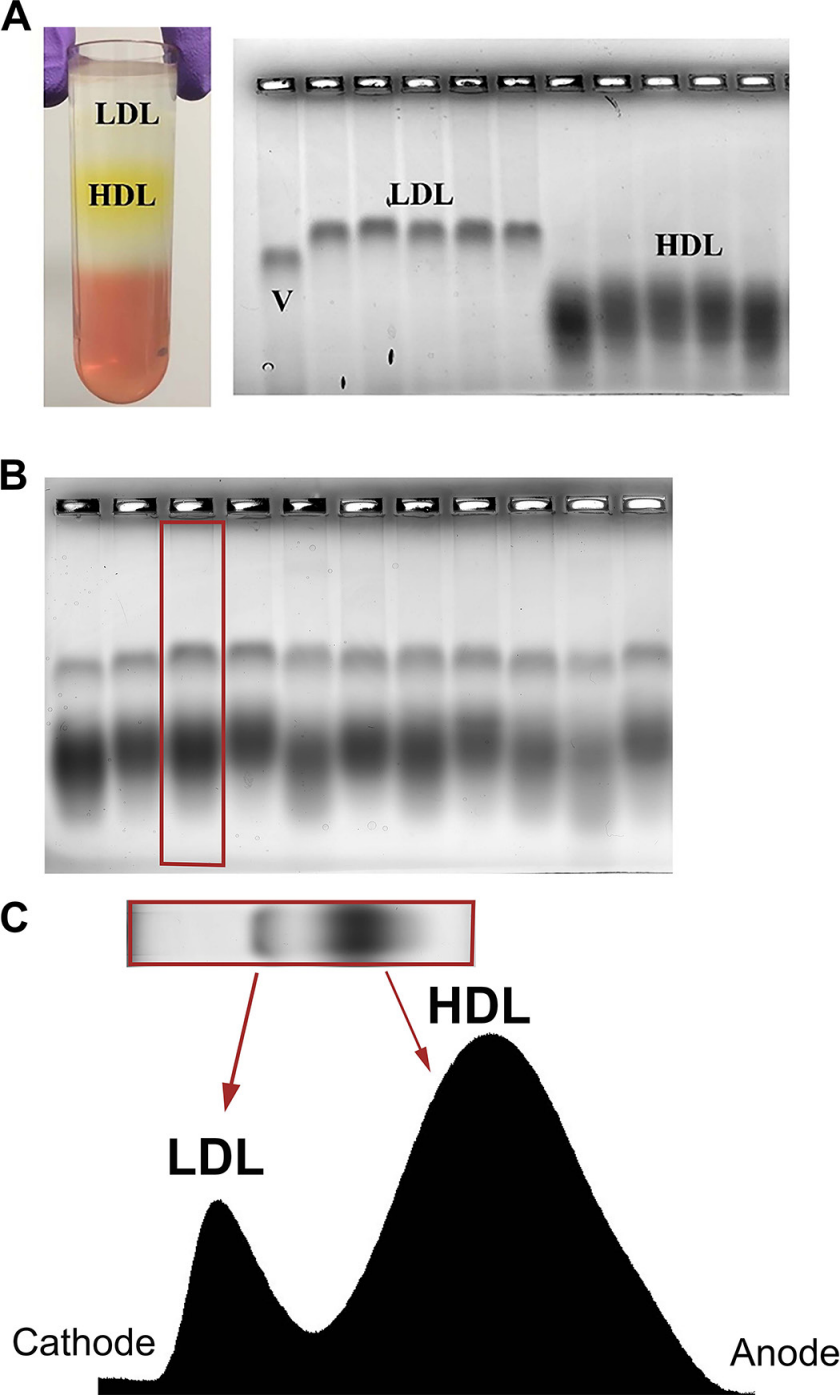
Lipoprotein fractions isolated by ultracentrifugation subjected to agarose electrophoresis to confirm the migration patterns in the gel and an example of a densitometry plot. Ultracentrifuged bovine plasma showing separation of low-density lipoprotein (LDL) and high-density lipoprotein (HDL; A, left) and validation of LDL and HDL migration pattern in agarose electrophoresis (A, right). The gel, stained with Sudan black B, depicts VLDL (V) pooled from 5 cows (due to very low concentrations in each individual cow) and samples from 5 individual bovine plasma samples separated by ultracentrifugation and electrophoresed after dialysis to remove excess salt. Example agarose gel showing lipoproteins from 16 cows that were electrophoresed and stained as described (B). Below the gel is the corresponding densitometry plot used to calculate area under the curve for each peak, which are then transformed to the percent of total lipoprotein (C).

Chemicals required for ultracentrifugation were all purchased from Sigma-Aldrich. Stock density gradient solutions were made as follows: 1.006 g/dL = 8.766 g of NaCl in 1 L of water with 1% (wt/vol) disodium EDTA, 1.346 g/dL = 153 g of NaCl, and 354 g of KBr in 1 L of water with 1% (wt/vol) disodium EDTA. Working gradient solutions were prepared by mixing these stock solutions using the equation: Df = (d1 × v1) + (d2 × v2)/v1 + v2, where Df is the final density, d1 and d2 are component densities, and v1 and v2 are the respective volumes. To prepare the plasma for ultracentrifugation; 13 mL of EDTA-anticoagulated plasma was brought to a density of 1.34 g/dL using solid KBr, overlaid with 4 mL of d = 1.18 g/dL solution, followed by 6 mL of d = 1.091 g/dL solution, and then 6 mL of d = 1.063 g/dL KBr, NaCl EDTA, followed by 2 mL of d = 1.006 g/mL. The gradient was centrifuged at 126,000 × *g* for 22 h at 4°C in a SW 32Ti swinging bucket rotor, slow acceleration, and no applied brake. The fractions were separated by sequential aspiration beginning at the top of the tube: 2 mL = VLDL, 4 mL = LDL, 4 mL LDL/HDL interface (discarded), 8 mL HDL (11–18 mL).

Passing-Bablok regression analysis was used to compare analytes across methods, followed by generation of Bland-Altman difference plots for the LDL and HDL percentages only, for data visualization purposes only. Simple linear regression was used if the correlation was insufficient to generate a Passing-Bablok plot (Prism GraphPad Software version 8).

Total cholesterol values in the samples ranged from 1.4 to 6.66 mmol/L (54 to 257 mg/dL) with a median value of 2.77 mmol/L (107 mg/dL). Sixteen samples had total cholesterol values less than 2.59 mmol/L (100 mg/dL), 28 samples had values between 2.59 and 3.88 mmol/L (100 and 150 mg/dL), and 12 samples had values exceeding 3.88 mmol/L (150 mg/dL). Samples with hemolysis were excluded. Precision of the manual electrophoresis procedure has been previously reported; with intraassay coefficients of variation (n = 40 replicates) of LDL and HDL equal to 11.4 and 2.27%, respectively (Behling-Kelly, 2016). The coefficients of variation (n = 21 replicates) for the Roche assay provided by the manufacturer are 1.3% and 1.2% for the low and high level human serum controls, respectively. Correlation between the 2 methods was poor HDL (Passing-Bablok regression line: y = 42.3 + 0.68x) and could not be calculated for LDL due to automated HDL values that were equal to, or higher than, the total cholesterol concentration in 25 of the 56 samples. Linear regression analyses are shown to allow visualization of the data (Figure 2). In all samples, LDL was detected by electrophoresis (average of 18% of all lipoproteins, SD = 0.8%, n = 56). Eighteen samples had TG concentrations above the reference interval and these samples had an average of 96% of the cholesterol measured as HDL by the automated method, and 78% of their lipoprotein content measured as HDL by electrophoresis. Triglyceride concentrations were moderately correlated with LDL percentage as determined by electrophoresis (r = 0.39).

**Figure 2:**
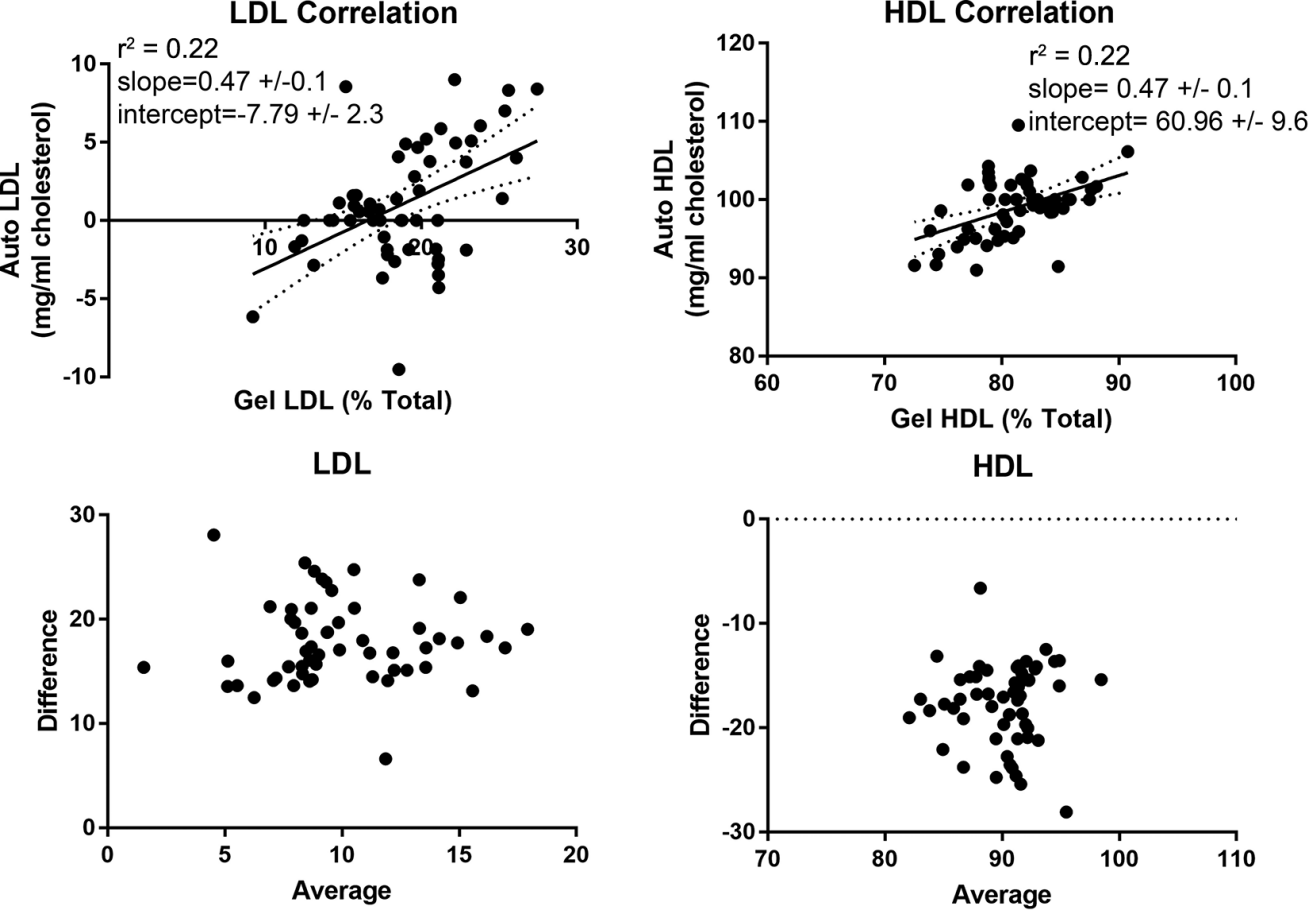
Linear regression (top row of panels) and Bland-Altman plots (lower row of panels) comparing low-density lipoprotein (LDL; right) and high-density lipoprotein (HDL; left) values using the automated wet chemistry method (Auto) and agarose electrophoresis (Gel).

Accurate evaluation of lipid metabolism in dairy cattle is essential to monitoring the metabolic state and health of both individual animals and herds. The use of correlational comparisons to validate assays is not sufficient, as correlations mask biases. Comparison of results across studies requires knowledge of the accuracy and biases inherent in each method used. Hence, there is a need to perform full validation studies and Bland-Altman comparisons when a method or assay developed for use on human samples is used in veterinary research (Jensen and Kjelgaard-Hansen, 2006). In this study, we demonstrated that the Roche HDL3 reagent performed poorly compared with electrophoresis when used to evaluate bovine HDL concentrations. In many instances, physiologically impossible results were generated. The relative proportions of LDL and HDL we measured are similar to those reported in other studies using similar electrophoretic methods (Crociati et al., 2017; Giordano et al., 2017). Our findings draw into question the accuracy of the Roche automated assay in quantifying bovine lipoprotein fractions, and these findings may extend to other wet chemistry methods.

There are several biochemically based reasons to explain poor transference of lipoprotein quantification by wet chemistry methods across species. Bovine lipoproteins are not identical in composition to human lipoproteins. The majority of bovine HDL spans a density range of 1.063 to 1.125 g/mL, which is equivalent to the density range of one type of HDL (HDL_2_) in people. The bovine has only trace amounts of HDL in the range of the major human HDL subtype, HDL_3_ (Dryden et al., 1971; Jonas, 1972; Stead and Welch, 1976). Bovine HDL has more surface-exposed protein, and a higher proportion of cholesterol ester than human HDL and specific bovine LDL subtypes are higher in phospholipid content compared with human LDL (Jonas, 1972; Stead and Welch, 1975). Given the unique chemical properties of bovine lipoproteins, it is not surprising that Roche reagents developed to react with human HDL generate questionable results in this species. Our electrophoretic results are more concordant with studies that used Bayer's Technicon RA 1000 (Stead and Welch, 1976). Perhaps the Bayer reagents perform adequately with bovine lipoproteins. Studies would be required to validate or refute this hypothesis.

A limitation of the current study is the relative lack of extreme values. This is partially due to the biology of dairy cattle, which consistently tend to be a HDL-rich species with a relatively small amount of LDL. Additionally, this study was performed with the third generation Roche reagent, and a fourth generation reagent is now commercially available that may react differently with the lower density lipoproteins in cattle. Measurement of animal lipoproteins is a challenge using currently available reagents. The methodology for the measure of total cholesterol and triglycerides employs the enzymatic breakdown of the lipoproteins and detection of freed cholesterol and the glycerol backbone, respectively. Therefore, these assays are broadly useful across species barriers. Electrophoretic methods, and other means of physical separation of lipoproteins, tend to be more laborious and costly, but do not rely on potentially species-specific reagents. Studies comparing between specific groups of animals, or across disease states, but employing consistent methodology are less subject to the confounding effects of assay inaccuracy than those seeking to define a diagnostically useful threshold concentration of lipoproteins. Assay limitations and the need for cross-species validation should be considered in study design and execution.

## References

[bib1] Behling-Kelly E. (2016). Comparison of 2 electrophoretic methods and a wet-chemistry method in the analysis of canine lipoproteins. Vet. Clin. Pathol..

[bib2] Chapman M.J. (1980). Animal lipoproteins: Chemistry, structure, and comparative aspects. J. Lipid Res..

[bib3] Chapman M.J. (1986). Comparative analysis of mammalian plasma lipoproteins. Methods Enzymol..

[bib4] Crociati M., Sylla L., Floridi C., Comin A., Fruganti G., Monaci M., Stradaioli G. (2017). Influence of lipoproteins at dry-off on metabolism of dairy cows during transition period and on postpartum reproductive outcomes. Theriogenology.

[bib5] Dryden F.D., Marchello J.A., Adams G.H., Hale W.H. (1971). Bovine serum lipids. II. Lipoprotein quantitative and qualitative composition as influenced by added animal fat diets. J. Anim. Sci..

[bib6] Evans L., Patton S., McCarthy R.D. (1961). Fatty Acid Composition of the Lipid Fractions from Bovine Serum Lipoproteins. J. Dairy Sci..

[bib7] Giordano A., Rossi G., Probo M., Moretti P., Paltrinieri S. (2017). Colorimetric and electrophoretic evaluation of lipoprotein fractions in healthy neonatal calves: Comparison with results from adult cows and from calves with inflammatory conditions. Res. Vet. Sci..

[bib8] Gleockler D.H., Ferreri L.F., Flaim E. (1980). Lipoprotein patterns in normal lactating Holstein cows bled at various times: Effects of milking. Exp. Biol. Med. (Maywood).

[bib9] Gross J.J., Kessler E.C., Albrecht C., Bruckmaier R.M. (2015). Response of the cholesterol metabolism to a negative energy balance in dairy cows depends on the lactational stage. PLoS ONE.

[bib10] Jensen A.L., Kjelgaard-Hansen M. (2006). Method comparison in the clinical laboratory. Vet. Clin. Pathol..

[bib11] Jonas A. (1972). Physicochemical properties of bovine serum high density lipoprotein. J. Biol. Chem..

[bib12] Jonas A. (1973). Location of aromatic amino acid residues in bovine serum high-density lipoprotein. Biochemistry.

[bib13] Kessler E.C., Gross J.J., Bruckmaier R.M., Albrecht C. (2014). Cholesterol metabolism, transport, and hepatic regulation in dairy cows during transition and early lactation. J. Dairy Sci..

[bib14] Mohebbi-Fani M., Nazifi S., Shekarforoush S.S., Rahimi M. (2006). Effect of monensin on serum lipoproteins, triglycerides, cholesterol and total lipids of periparturient dairy cows. Vet. Res. Commun..

[bib15] Nichols A.V. (1967). Human serum lipoproteins and their interrelationships. Adv. Biol. Med. Phys..

[bib16] Saleem S., Heuer C., Sun C., Kendall D., Moreno J., Vishwanath R. (2016). Technical note: The role of circulating low-density lipoprotein levels as a phenotypic marker for Holstein cholesterol deficiency in dairy cattle. J. Dairy Sci..

[bib17] Stead D., Welch V.A. (1975). Lipid composition of bovine serum lipoproteins. J. Dairy Sci..

[bib18] Stead D., Welch V.A. (1976). Determination of physical properties of bovine serum lipoproteins by analytical ultracentrifugation. J. Dairy Sci..

[bib19] Turk R., Juretic D., Geres D., Turk N., Rekic B., Simeon-Rudolf V., Robic M., Svetina A. (2005). Serum paraoxonase activity in dairy cows during pregnancy. Res. Vet. Sci..

[bib20] Turk R., Podpečan O., Mrkun J., Kosec M., Flegar-Meštrić Z., Perkov S., Starič J., Robić M., Belić M., Zrimšek P. (2013). Lipid mobilisation and oxidative stress as metabolic adaptation processes in dairy heifers during transition period. Anim. Reprod. Sci..

[bib21] Wu J., Liu J., Wang D. (2020). Effects of body condition on the insulin resistance, lipid metabolism and oxidative stress of lactating dairy cows. Lipids Health Dis..

